# Palliative care in Uganda: quantitative descriptive study of key palliative care indicators 2018-2020

**DOI:** 10.1186/s12904-022-00930-7

**Published:** 2022-04-22

**Authors:** Ainur Kagarmanova, Mark Donald Mwesiga, Matthew L. Sisk, Cynthia Kabagambe, Sheba Nyakaisiki, Tom Marentette, Lacey N. Ahern

**Affiliations:** 1 Eck Institute for Global Health, University of Notre Dame, Notre Dame, IN USA; 2 Palliative Care Association of Uganda, Kampala, Uganda; 3 Lucy Family Institute for Data and Society, University of Notre Dame, Notre Dame, IN USA; 4 Uganda Martyrs University, Kampala, Uganda; 5 Office of Information Technologies, University of Notre Dame, Notre Dame, IN USA; 6 Hospice Foundation/Global Partners in Care, Mishawaka, IN USA

**Keywords:** Palliative care, mHealth, Morphine, Uganda

## Abstract

**Background:**

The first and most recent nationwide audit of palliative care services in Uganda was conducted in 2009. Since then, Uganda has made great strides in palliative care development, including policy, education, and services implementation. This study provides an overview of the availability of palliative care services in the country and the challenges and gaps in Uganda prior to the global COVID-19 pandemic. This lays the foundation for better understanding the challenges and changes needed to support palliative care development and access in the wake of the pandemic.

**Methods:**

We conducted a descriptive quantitative study of secondary data on nationwide morphine distribution, collated a list of accredited facilities, and analyzed key palliative care indicators collected through the mHealth surveillance project present at a subset of accredited facilities. Descriptive statistical analysis involved non-parametric tests using SPSS, mapping geographical distribution of available palliative care services using Geographic Information Systems software, and identification of challenges from the subset of accredited facilities.

**Results:**

There were 226 accredited palliative care facilities across Uganda’s 135 districts in 2020. Thirty districts lacked any accredited palliative care facility. The estimated population coverage was 88.5%. The majority (68.1%) of accredited facilities were public, and private facilities received slightly more pain-relieving morphine. There was an alternating trend in the volumes of morphine delivered to public and private facilities. More than a third of the patients were diagnosed with non-communicable diseases, highlighting their significance alongside cancer and HIV/AIDS as conditions requiring palliative care. Palliative care accredited facilities offered six types of services: outreach, home visits, psychosocial, legal, bereavement, and spiritual support, but only for an average of 7 months a year due to lack of facilitation and transportation.

**Conclusion:**

Palliative care in Uganda developed in quality, volume, and geographic coverage since 2009. The shift in palliative care patients’ primary diagnosis from HIV/AIDS to non-communicable diseases marks an important epidemiologic transition. Although accredited facilities are present in most administrative districts, more research is needed to evaluate the actual accessibility of these services. The existing services, both private and public, are limited by the amount of pain-relieving morphine, financial and transport resources. More quality data collected on key palliative care indicators is needed into geographical accessibility of palliative care services, morphine availability trends, and patient diagnoses in order to improve the provision of palliative care in Uganda.

**Supplementary Information:**

The online version contains supplementary material available at 10.1186/s12904-022-00930-7.

## Background

Globally in 2020, only 7 million people - approximately 12% of the 56.8 million people in need of palliative care - received it [1]. Low- and middle-income countries account for 76% of the global need in palliative care [1]. For countries to meet this demand for essential care, it is recommended to integrate palliative care services into existing healthcare systems and track them with proper data and indicators to ensure quality of services [2–4]. Quality data directly affects the provision of quality palliative care as it is a major factor in financial decision-making by governments, third-party payers, and insurers [5]. Capturing patient-level data is a necessary condition in identifying and addressing gaps in palliative care [6]. One method of ensuring quality data collection is using mobile phones and tablets as tools to further advance both data collection and service delivery in healthcare [7, 8]. This is especially relevant in Africa where, on average, there are 76 mobile-cellular subscriptions per 100 people [9]. Leveraging this technology, mobile health (mHealth) interventions in the form of short message service (SMS) notifications have been used to increase appointment adherence and rapport with patients in oncologic settings of rural Uganda [10]. To facilitate collaboration between current mHealth activities, the African Palliative Care Association (APCA) has initiated a mHealth Research Network [11].

### Palliative care development in Uganda

A systematic approach to palliative care in Uganda was established in 1993 by Dr. Anne Merriman and Fazal Mbaraka as a model for African palliative care [12]. The high prevalence of HIV/AIDS and cancer drove the development of palliative care in Uganda [12]. The majority (85%) of the palliative care services in Uganda are provided by public hospitals, and there are also 77 private hospices or palliative care services in private hospitals in the country [13]. The overwhelming amount of funding for palliative care, 94%, comes from external donors [14]. In addition to this external funding, there are many champions of palliative care development in the government and private sector in Uganda who have worked to increase education and awareness of the need for palliative care as well as ensure access to essential medicines. Medicines, such as opioids, are critical to relieve pain and other symptoms in palliative care provision [4, 12]. Oral morphine, produced from imported morphine powder, was introduced to Uganda in 1993 as a cheap and effective option for pain relief [12].

Advocacy by the Palliative Care Association of Uganda (PCAU) and Hospice Africa Uganda (HAU) contributed to official registration of morphine, allocation of state funds and free provision of oral morphine to patients [14]. Imported powdered morphine is used to produce oral morphine of two concentrations - 5 mg/ml green and 50 mg/ml red morphine. Morphine is distributed in Uganda via two suppliers - National Medical Stores (NMS) which supplies public facilities and Joint Medical Stores (JMS) which supplies private facilities [15, 16]. The International Narcotics Control Board (INCB) recognizes oral morphine as a controlled substance and this means governments must license, supervise, and report to INCB the production and distribution of all morphine [17]. In Uganda, this issue is addressed by tracking morphine distribution by suppliers and requiring receiving facilities to be accredited by PCAU. Accreditation status depends on three criteria that must be present at a facility: 1) properly trained palliative care staff, 2) double-locked morphine storage cabinet, 3) proper morphine tracking records [3]. Uganda was the first country to allow nurses and clinical officers with specialized training to prescribe opioid analgesics to increase accessibility and uptake of morphine [18]. Because nurses provide a significant amount of palliative care across the globe, providers in many other countries aspire to implement similar legislation [19].

### Palliative care data in Uganda

In Uganda, it is estimated that only 10% of the need for palliative care is met. Through their national health management information system (HMIS), the Ministry of Health (MoH) collects facility-level data on number of patients seen in pain and amount of morphine prescribed [20]. There are national and regional efforts to gather better data on palliative care to fully describe the current situation of palliative care services in the country [21]. On the national level, PCAU collects data on facility accreditation status and morphine distribution. In 2015, PCAU, the Center for Hospice Care/Hospice Foundation and the University of Notre Dame piloted an mHealth surveillance project to collect key palliative care indicators via mobile phones and address the lack of quality data at a subset of healthcare facilities across the country [15]. The project collects palliative care data at 20 facilities geographically dispersed throughout the country. Building on this project, PCAU is now working in partnership with the MoH to integrate palliative care data collection into the national health information system so Uganda can have a consistent and reliable source of palliative care data. In March of 2021, the MoH issued a directive for hospitals to allocate space for palliative care services, re-affirming the need in quality palliative care collection nationwide [22].

### Research aims

Given political, social, and technological developments since the last audit was conducted in 2009, this study offers a timely and comprehensive assessment of the current situation of palliative care in Uganda [21]. The aim of this study is to describe the palliative care situation in Uganda in terms of availability of morphine and services by district, availability of personnel at facilities, patient demographics and diagnoses contributing to the palliative care burden. By assessing aggregate data from the mHealth surveillance project facilities, along with national morphine distribution and accreditation data, this study provides an in-depth analysis of key palliative care indicators on the regional and national levels and offers maps visualizing geographic distribution of palliative care services.

## Methods

We conducted a descriptive quantitative study of data collected by PCAU. The analyzed data were obtained from three sources: the list of accredited facilities updated as of January 2020, morphine distribution data for 77 facilities for 2019, and mHealth surveillance survey (afterwards referred to as survey) responses from 20 facilities from January 2018 to February 2020.

### National-level data

The indicators analyzed at national level were: number of accredited palliative care facilities, number and type of services that received morphine, volume of morphine distribution, and population coverage. The most recent national census data is from 2014 and were used for the population coverage assessment. The districts in Uganda are grouped into ten subregions [23]. Due to frequent changes in the number of districts in Uganda, these larger subregional administrative divisions were used as a more permanent and comparable option.

### mHealth surveillance survey data

The survey consists of 75 questions and collects data from 20 facilities on: number of medical personnel involved in palliative care provision, morphine availability by type, palliative care patient diagnosis and mortality, palliative care services offered at the facility and challenges in offering these services. The 20 participating facilities represent both urban and rural locations, private and public facilities and are spread across ten of Uganda’s subregions. To make measurements comparable between facilities, monthly averages of numerical indicators (i.e., number of patients, volume of morphine used) were used.

### Statistical tests

The geographic distribution of accredited facilities was mapped using ArcGIS Desktop 10.7. The palliative care situation in the country was described in terms of availability of morphine and accredited services by district, availability of personnel at facilities, patient demographics, and diagnoses. The relationship between the facility type and palliative care indicators collected was examined with Wilcoxon-Rank Sum test, Spearman’s Rank correlation, and Kruskal-Wallis test. *P*-values below 0.05 were considered statistically significant. One survey facility was excluded from the analysis because of an absence of data.

### Ethical considerations

The study did not involve recruitment of vulnerable populations, collection of personal medical records, or any other sensitive information. No personal identifiers were collected with any data. All data is anonymized and were aggregated at the facility level for analysis. The data were stored on a password-protected personal computer of the principal investigators. Authorized members of the research team from PCAU, the University of Notre Dame, and Uganda Martyrs University had access to the data. The study was approved by the University of Notre Dame Institutional Review Board (NDIRB), protocol number is 20-05-6066 and Hospice Africa Uganda Research Ethics Committee (HAUREC) reference number HAUREC-083-20. The study adheres to all human data institutional guidelines of NDIRB and HAUREC.

## Results

### National level data

A total of 226 accredited palliative care facilities^1^ were identified in Uganda, and of them, 154 (68.1%) were public facilities and 72 (31.9%) were private facilities. Only 77 (34.1%) of accredited facilities reported receiving pain-relieving morphine in 2019 (Table 1). A full list of those accredited facilities who received no morphine in 2019 can be found in Additional file 1.

**Table 1:** Palliative care accreditation and morphine use in Uganda by subregions, 2019 (*n* = 226)

Subregion	Number of accredited facilities		
	Received morphine	Didn’t receive morphine	Total
Acholi	4 (30.8%)	9 (69.2%)	13
Central	27 (40.9%)	39 (59.1%)	66
East Central	6 (30.0%)	14 (70.0%)	20
Elgon	8 (42.1%)	11 (57.9%)	19
Karamoja	2 (33.3%)	4 (66.7%)	6
Lango	2 (22.2%)	7 (77.8%)	9
Southwestern	11 (33.3%)	22 (66.7%)	33
Teso	5 (31.3%)	11 (68.8%)	16
West Nile	4 (25.0%)	12 (75.0%)	16
Western	8 (28.6%)	20 (71.4%)	28
**All Uganda**	**77 (34.1%)**	**149 (65.9%)**	**226 (100%)**

There were 4 unaccredited facilities that received morphine in Central subregion

**Fig. 1:**
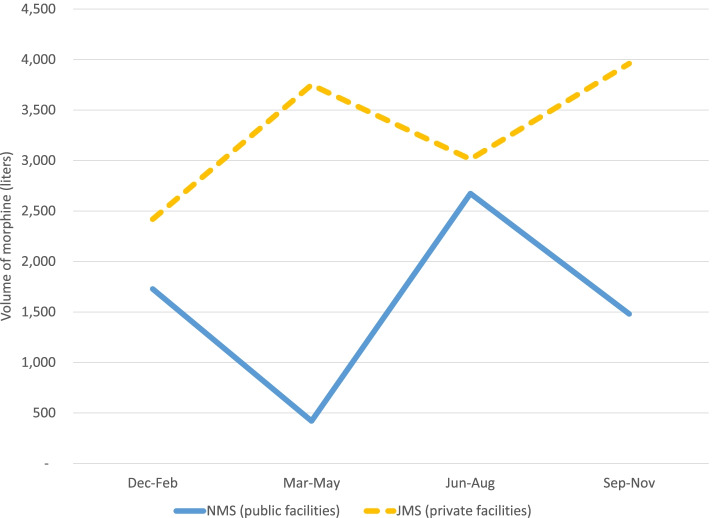
Seasonal distribution of the morphine volume by facility type

In addition to the 77 accredited facilities receiving morphine in 2019, four other facilities who were not accredited by PCAU received morphine. JMS distributed a slightly larger amount of morphine (100,627,500 ml) and more frequent deliveries of morphine than NMS that delivered a total of 9,120,000 ml. There was an inverse relationship in the volumes of morphine supplied by JMS and NMS that was statistically significant in May (*p* < 0.001), June (*p* = 0.003), September (*p* = 0.043), and December (*p* = 0.045) of 2019. The detailed morphine volumes by month provided to the mHealth survey facilities are listed in Additional file 2. In the months when the difference in the volume of green morphine delivered was statistically significant, NMS orders were on the rise when JMS orders decreased, and vice versa (Fig. 1).

#### Geographical distribution of palliative care services

**Fig. 2:**
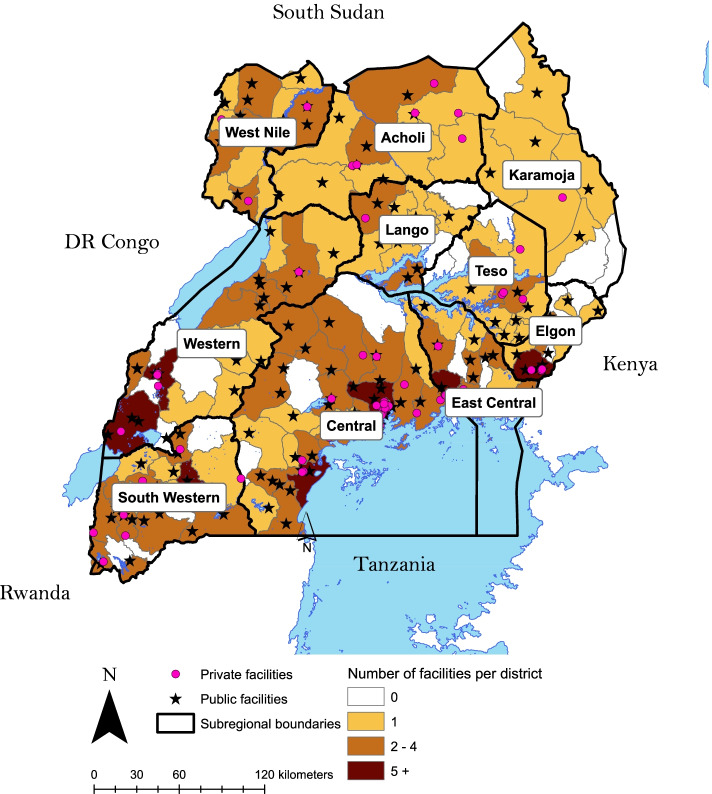
Map of accredited palliative care facilities in Uganda as of January 2020

Accredited palliative care facilities were present in all ten of Uganda’s subregions, but 30 of the country’s 135 (at the time of analysis) districts had no palliative care facilities (Fig. 2). Almost a third of the districts in Elgon, a subregion in Eastern Uganda, had no palliative care facilities. The list of districts with no accredited palliative care facilities is provided in Additional file 3.

#### Population coverage with palliative care services

**Fig. 3:**
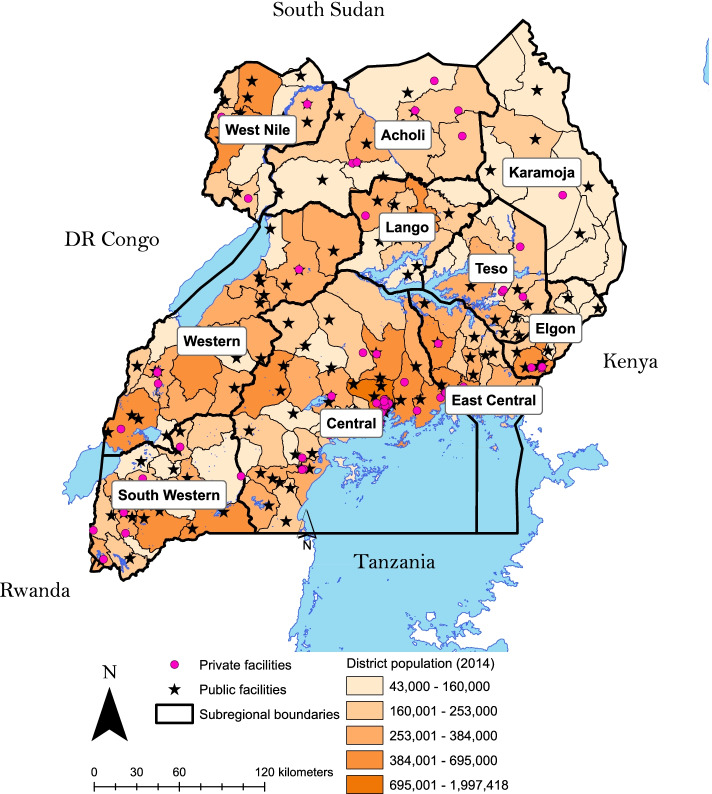
Population coverage of palliative care services

To evaluate the population coverage of palliative care services, we mapped the 2014 Census data available at the subcounty (administrative level 5) level on a 2020 administrative districts map [24]. These data were used to allocate the population of each 2014 subcounty to a 2020 district. Since the internal boundaries did not change, it is reasonable to use these data for comparisons over time. Based on the 2014 census data, 88.5% of people in Uganda live in a current district with at least one palliative care service available, whereas 18.0% of people live in a district with five or more palliative care services (Fig. 3, Additional file 4). Districts with the most palliative care services were Kampala [18], Wakiso [12], Tororo [6]; Jinja, Kabarole, Masaka, Mbarara, Ntungamo and Rukungiri each had five accredited facilities.

### Data collected from mHealth surveillance survey

Twenty facilities participated in the mHealth surveillance survey. One is excluded from analysis because of insufficient data, leaving a sample size of 19. These facilities represent 16 districts and 10 subregions of Uganda (Table 2).

**Table 2:** Type and location of the mHealth surveillance survey participating facilities

Facility	Type	District	Subregion
Adjumani Hospital	Public	Adjumani	West Nile
Arua Regional Referral Hospital (RRH)	Public	Arua	West Nile
Fort Portal RRH	Public	Kabarole	Western
Gulu RRH	Public	Gulu	Acholi
Hospice Africa Uganda	Private	Kampala	Central
Hospice Jinja	Private	Jinja	East Central
Hospice Tororo	Private	Tororo	Elgon
Kabale RRH	Public	Kabale	Southwest
Kawempe Home Care	Private	Kampala	Central
Kisoro Hospital	Public	Kisoro	Southwest
Kitovu Home Care	Private	Masaka	Central
Little Hospice Hoima	Private	Hoima	Western
Masaka RRH	Public	Masaka	Central
Matany Hospital	Private	Napak	Karamoja
Mbale RRH	Public	Mbale	Elgon
Mobile Hospice Mbarara	Private	Mbarara	Southwest
Soroti RRH	Public	Soroti	Teso
St. Francis Naggalama Hospital	Private	Mukono	Central
St. Mary Hospital Lacor	Private	Gulu	Acholi

Hospice Africa Uganda, Hospice Tororo, Kawempe Home Care, Kitovu Home Care, Little Hospice Hoima, Mobile Hospice Mbarara, and Rays of Hope Hospice Jinja are stand-alone hospices. All others are palliative care units within a hospital/facility

#### Distribution of morphine

Based on the survey data from 19 facilities, private facilities on average received higher volumes of green morphine (5 mg/ml); although this finding was not statistically significant (Table 3).

**Table 3:** Morphine distribution in mHealth survey participating facilities, 2019

Indicator	Public facilities (***n*** = 9)	Private facilities (***n*** = 10)	Total (***n*** = 19)
**Average volume of morphine received per month, ml**	**30,264**	**41,288**	**35,776 [100%]**
Green morphine (5 mg/ml)	30,264	39,234	34,749 [97.1%]
Red morphine (50 mg/ml)	0	2054	2054 [5.7%]
**Average number of stock-out days per month**	**9**	**18.5**	**13.75**
Green morphine (5 mg/ml)	9	13	11
Red morphine (50 mg/ml)	N/A	24	24

#### Challenges in accessing morphine

All 19 facilities reported experiencing issues with receiving morphine. The most cited challenges among public facilities had to do with a supplier, while private facilities cited a non-specified ‘other’ category as the most common one (Table 4). It is worth noting that NMS delivers morphine to facilities, whereas JMS requires facilities to pick up morphine from them. The challenge “morphine not requisitioned” may mean a facility did not place an order in time, and this was more common for public facilities.

**Table 4:** Number of times a challenge in accessing morphine was reported during 2019

	Public (*n* = 112)	Private (*n* = 169)	Total (*n* = 281)
**Issue at facility**	**4 [3.6%]**	**3 [1.8%]**	**7 [2.5%]**
Prescriber not there	1 [0.9%]	0 [0.0%]	1 [0.4%]
Morphine not requisitioned	3 [2.7%]	3 [1.8%]	6 [2.1%]
**Issue with supplier**	**7 [6.3%]**	**8 [4.8%]**	**15 [5.3%]**
Not delivered by NMS	6 [5.4%]	NA	6 [2.1%]
Not picked up by JMS	NA	4 [2.4%]	4 [1.4%]
Supplier out of stock	1 [0.9%]	4 [2.4%]	5 [1.8%]
Morphine was expired	0 [0.0%]	0 [0.0%]	0 [0.0%]
**Other**	**6 [5.4%]**	**10 [5.9%]**	**16 [5.7%]**

#### Patient demographics and diagnoses

On average private facilities treated a larger number of palliative care patients. Public facilities treated a larger proportion (27.6%) of child patients than private facilities (10.0%). The difference in the number of patients across demographic groups and the number of deaths (Table 5) was not statistically significant.

**Table 5:** Palliative care patient characteristics seen at 19 facilities (January 2018 to February 2020)

Indicator	Public facilities (***n*** = 9)	Private facilities (***n*** = 10)	Total (***n*** = 19)
Average number of months reported in the mHealth survey^a^	16.3	16.6	16.5
**Patients**			
**Average number of patients per month**	**76 [29.6%]**	**181 [70.4%]**	**128.5 [100%]**
Male adults	22 [8.6%]	57 [22.2%]	39.5 [30.7%]
Female adults	34 [13.2%]	106 [41.3%]	70 [54.5%]
Male children	10 [3.9%]	10 [3.9%]	10 [7.8%]
Female children	11 [4.3%]	8 [3.1%]	9.5[7.4%]
**Average number of deaths per month**	**2.8 [19.6%]**	**11.3 [80.4%]**	**7.1 [100%]**
Adults	2.4 [17.3%]	10.2 [73.4%]	6.3 [89.4%]
Children	0.4 [2.9%]	1.1 [7.9%]	0.8 [10.6%]
**Total number of patient visits**	**29,841 [50.5%]**	**29,226 [49.5%]**	**59,067 [100%]**
HIV/AIDS	5391 [9.1%]	4628 [7.8%]	5009.5 [17.0%]
Cancer	4272 [7.2%]	18,471 [31.3%]	11,371.5 [38.5%]
HIV/Cancer comorbidity	454 [0.8%]	3033 [5.1%]	1743.5 [5.9%]
Other^b^	19,724 [33.4%]	3094 [5.2%]	11,409 [38.6%]
**Services**			
**Average monthly number of HCWs**	**16**	**18**	**34 [100%]**
Doctors	3	3	6 [17.6%]
Nurses	6	6	12 [35.3%]
Others	7	9	16 [47.1%]

^a^ Analysis spans 26 months of data but several facilities do not have data submissions from every month due to challenges with staff, internet, etc. Additionally, the data sample used in this analysis ends at the beginning of the COVID-19 Pandemic (March 2020) because of global changes in the need and resources for palliative care

^b^Top three other conditions include hypertension, arthritis, and pains

Cancer was the top diagnosis of patients seen (38.5%) followed by HIV/AIDS (17.0%). Cancer types are provided in Additional file 5. Palliative care teams are caring for patients with a wide variety of diagnoses; 38.6% of visits to palliative care facilities were attributed to more than 50 other conditions with hypertension, arthritis, and pains reported the most (Table 5, Additional file 6). Although the total number of visits was approximately the same in private and public facilities, percentage-wise public facilities saw more patients with other conditions (33.4%, compared to 5.2% at private facilities) while private facilities saw more cancer patients (31.3% vs 7.2% at public facilities).

#### Availability of palliative care staff and services

As shown in Table 5, public facilities had a larger total number of personnel directly involved in palliative care per patient than private ones (*p* = 0.003). Similarly, the number of involved doctors was also larger in public facilities (*p* = 0.042). On average, both types of facilities offer at least one out of six possible types of palliative care services throughout the year. Psychosocial, bereavement, spiritual support and home visits appeared to be most likely to be available in public and private facilities (Table 6).

**Table 6:** Average number of months palliative care services offered at mHealth facilities by type per year

Service	Public facilities (***n*** = 9)	Private facilities (***n*** = 10)
Outreach	7.1	7.5
Psychosocial support	7.2	7.4
Bereavement support	7.2	7.5
Legal support	7.1	7.5
Home visits	7.2	7.5
Spiritual support	7.2	7.5

The average number of personnel per patient by health care worker type and facility were correlated to the average number of months a palliative care service was offered at a facility using the Spearman’s Rank correlation. Home visits, outreach, bereavement, legal, and spiritual services did not have a statistically significant correlation with the average number of any type of HCWs. There was a weak, negative correlation between the frequency of psychosocial services offered and the average number of other HCWs involved, which was statistically significant (*p* = 0.04).

## Discussion

This study provides a description of palliative care availability throughout Uganda with a deeper look at providers, services and patient demographics and diagnoses at a subset of facilities prior to the COVID-19 pandemic. This is the first study to comprehensively map palliative care services and morphine use across Uganda.

A seemingly widespread population coverage with palliative care facilities of 88.5% may not capture the whole picture of access to such facilities by patients. Living in a district with an accredited palliative care facility does not necessarily equal accessibility and availability of the service, as it may be hindered by transportation challenges and high medical costs. In Uganda, cancer patients cited lack of money and transportation as major reasons for delaying treatment or missing appointments; some patients had to travel between 13 and 212 km to a medical facility [25]. In another study, 46% of HIV/AIDS patients reported difficulty traveling to a hospital as a main barrier to accessing care, while 23% reported that they cannot afford medical care [19]. Even though more than 80% of people in Uganda lived within 5 km of a health facility, many preferred to travel further distances to access private health centers due to their perceived better quality [26]. There are no studies evaluating geographical access to palliative care services in Uganda. Geographical access has been estimated for malaria patients using the cumulative case ratio method of defining the hospital’s catchment area [27]. More complex geographical analysis was beyond the scope of this study but is recommended as an important follow up in determining accessibility of palliative care services. A similar approach could be taken to analyze access to palliative care facilities and combined with data on outreach and home visit services from these facilities. All mHealth survey facilities in our study provided outreach and home visit services, though these were not offered consistently throughout the year, with transport and other costs noted as barriers to providing the services. It is important to track the extent of such services for a better understanding of palliative care coverage, especially in rural areas.

In 2013, Nabudere et al. stated cancer and HIV/AIDS patients contribute 80% of all palliative care patients in Uganda [20]. Our data from the mHealth survey showed that overall, 61.4% of patients were diagnosed with cancer, HIV/AIDS, or both, while the remaining 38.6% was attributed to other conditions including arthritis, hypertension, and cardiac disease. This supports other evidence that sub-Saharan Africa is undergoing an epidemiological transition, as the proportion of disability-adjusted life years contributed by non-communicable disease (NCDs) increased from 18.6% in 1990 to 29.8% in 2017 [28]. The top three contributors to the NCD burden across sub-Saharan Africa were other NCDs, such as congenital disorders, cardiovascular diseases, and neoplasms [28]. Palliative care is recognized by the World Health Organization as an essential component of a comprehensive response to NCDs [29]. Despite this, only 4% of African countries offer palliative care services to NCD patients as a part of primary care [30].

Due to the lack of epidemiologic data on NCDs burden among palliative care patients, governments and donors have invested in HIV/AIDS care, while other conditions requiring palliative care are prioritized lower [30]. This lack of funding may manifest as the lack of resources, including transport and staff. Our findings showed both private and public facilities reported lack of transport and facilitation as the challenges across the six types of services (bereavement, spiritual, psychosocial, legal, outreach, home visits) for both private and public facilities. Hence, it is important to increase awareness and advocacy of the universality of palliative care services to increase funding opportunities and address patient needs outside of HIV/AIDS care.

Although access to physical pain relief is only part of palliative care services, the availability of morphine remains an important indicator of palliative care services. The inverse relationship between the two national suppliers of morphine in Uganda may suggest NMS and JMS compete for morphine produced and available. There are more than double the number of public accredited facilities than private, but private facilities were supplied with more morphine. Past studies suggest this difference is due to higher uptake of private medical services by richer people [26, 31]. Our data did not allow for the analysis of patients’ socioeconomic status and distance to the private or public facilities. Private and public facilities in our mHealth survey cited the supplier stockouts and challenges with delivery or pick-up from NMS and JMS, respectively, as a major challenge in accessing the necessary volume of morphine. This again may indicate that transport challenges are experienced by suppliers and facilities and are hindering availability of palliative care. In the months of lower volume of morphine received, public facilities also reported a challenge of not ordering morphine on time which suggests the need for a more systematic approach to plan, order and manage morphine at the facility level.

### Limitations

The study analyzed data from a self-reported survey that introduces the possibility of human errors. The number of months reported in the survey varied from 5 to 26 over a 26-month period; therefore, the presented analysis may not be representative of all 19 facilities to the same extent. Challenges in accessing morphine were only assessed for facilities that received morphine at least once in 2019; however, the accredited facilities that did not receive morphine were not surveyed as part of this study and may indicate even more challenges. Accreditation data was last updated in January 2020, however the period for which accreditation status was granted for was not recorded. Hence, some facilities on the list may no longer meet the accreditation criteria nor be actively providing palliative care services. PCAU typically monitors facilities quarterly, usually by phone call and occasionally by site visit. But especially in public facilities, staff are frequently moved to other facilities and sometimes hard to track. Using morphine ordering as one means of continued monitoring of accredited facilities is an important tool for initial analysis of the status of service provision at these facilities.

Geographical coverage with palliative care services was implied from a presence of at least one palliative care service in a district. Since the straight-line distance or road network distance to a palliative care service was not considered, the actual accessibility may differ from that presented here.

## Conclusion

Palliative care was introduced to Uganda in 1993, and since then palliative care services provision has improved in quality, volume, and geographical coverage. Although accredited facilities are present in most administrative districts, more research is needed to evaluate the actual accessibility of these services, especially to rural residents. The existing services, both private and public, are limited by the amount of pain-relieving morphine, financial and transport resources. By aggregating morphine distribution data with the list of accredited facilities we were able to show there are many accredited facilities that do not regularly receive morphine; although the underlying reasons remain unclear, these facilities present an opportunity to better understand and address reasons that morphine may not be used when medically needed. The facilities primarily treat cancer, HIV/AIDS patients, and patients with other conditions such as hypertension, arthritis, and congestive cardiac failure. This indicates an important epidemiological transition, and the need for palliative care services to partner with organizations focused on treatment of NCD’s to increase access to those in need and ensure sustainability of palliative care services.

## Supplementary Information

**Additional file 1.** Accredited facilities that received no morphine in 2019. A full list of those accredited facilities in Uganda who received no morphine in 2019.

**Additional file 2.** Average monthly volumes (ml) of green morphine distributed to mHealth surveillance survey facilities in 2019. List of detailed morphine volumes by month.

**Additional file 3.** Districts with no accredited palliative care facility as of January 2020. List of districts with no accredited palliative care facilities as of January 2020.

**Additional file 4.** Number of people living in districts with palliative care facilities by Uganda’s subregions (2014 census). List of Uganda’s subregions and number of people living in districts by number of accredited palliative care facilities, indicating the need in palliative care services in number of people.

**Additional file 5.** Other cancers reported by the mHealth surveillance survey participating facilities. Types of cancer of patients seeking palliative care services.

**Additional file 6.** Other conditions mentioned by mHealth surveillance survey participating facilities. List of other conditions like hypertension, arthritis, and pain, reported the most when seeking palliative care.

## Data Availability

Data supporting the conclusions of this article are available in the CurateND, the University of Notre Dame’s institutional repository, [10.7274/r0-nqna-ka89]. National morphine data is third party data provided by the MOH to PCAU for data analysis. Authors can facilitate connection with the relevant persons in MOH for access to this data upon request.

## Abbreviations

APCA: African Palliative Care Association
HAU: Hospice Africa Uganda
HIV/AIDS: Human Immunodeficiency Virus/Acquired Immunodeficiency Syndrome
INCB: International Narcotics Control Board
JMS: Joint Medical Stores
mHealth: Mobile Health
MoH: Ministry of Health
NCD: Non-communicable diseases
NMS: National Medical Stores
PCAU: Palliative Care Association of Uganda
RRH: Regional Referral Hospital
SMS: Short Message Service

## Footnotes

1 Palliative care facilities include but are not limited to free standing hospices, hospices that are a part of public or NGO hospitals, any kind of other hospices or home care teams, support teams in hospitals, palliative care units, inpatient units in hospices, or paediatric palliative care hospices and services that are accredited by PCAU.
