# Supplementary material for: Palliative care in Uganda: quantitative descriptive study of key palliative care indicators 2018-2020
Source: BMC Palliat Care. 2022 Apr 22;21:55. doi: 10.1186/s12904-022-00930-7 (PMC9023726; doi:10.1186/s12904-022-00930-7)
Supplement: Supplementary file 1 — Additional file 1. Accredited facilities that received no morphine in 2019. A full list of those accredited facilities in Uganda who received no morphine in 2019. [file 12904_2022_930_MOESM1_ESM.docx]

Additional File 1

Accredited facilities that received no morphine in 2019

| 1 | Abim Hospital |
| --- | --- |
| 2 | Aboke Health Center IV |
| 3 | Aduku Health Center IV |
| 4 | Alebtong Health Center IV |
| 5 | Amolatar Health Center IV |
| 6 | Anaka Hospital |
| 7 | Angal Hospital |
| 8 | Anyeke Health Center IV |
| 9 | Apac General Hospital |
| 10 | Aputi Health Center III |
| 11 | Atiak Health Center IV |
| 12 | Awach Health Center IV |
| 13 | Bihimba Health Center IV |
| 14 | Bishop Caesar Asili Memorial Hospital |
| 15 | Budadiri Health Center IV |
| 16 | Budaka Health Center IV |
| 17 | Budondo Health Center IV |
| 18 | Bududa Hospital |
| 19 | Bufumbo Health Center IV |
| 20 | Bugamba Health Center IV |
| 21 | Bugangari Health Center IV |
| 22 | Bugembe Health Center IV |
| 23 | Bugobelo Health Center IV |
| 24 | Buhimba Health Center IV |
| 25 | Bukasa Health Center IV |
| 26 | Bukedea Health Center IV |
| 27 | Bukomero Health Center IV |
| 28 | Bukono Health Center IV |
| 29 | Bukulula Health Center IV |
| 30 | Bukwo Hospital |
| 31 | Bulisa Health Center IV |
| 32 | Bumanya Health Center IV |
| 33 | Bundibugyo Hospital |
| 34 | Bushenyi Health Center IV |
| 35 | Busia Health Center IV |
| 36 | Butabika National Referral Hospital |
| 37 | Butebo Health Center IV |
| 38 | Butenga Health Center IV |
| 39 | Buwambo Health Center IV |
| 40 | Buwasa Health Center IV |
| 41 | Bwera Hospital |
| 42 | Bwijanga Health Center IV |
| 43 | Bwizibwera Health Center IV |
| 44 | China-Uganda Friendship Hospital Naguru |
| 45 | Comboni Hospital |
| 46 | Dabani Hospital |
| 47 | Dwoli Health Center III |
| 48 | Entebbe Regional Referral Hospital |
| 49 | General Military Hospital Bombo |
| 50 | International Hospital Kampala |
| 51 | Ishaka Hospital |
| 52 | Ishongororo Health Center IV |
| 53 | Itojo Hospital |
| 54 | Kaabong General Hospital |
| 55 | Kabarole Hospital |
| 56 | Kabuyanda Health Center IV |
| 57 | Kakumiro Health Center IV |
| 58 | Kakuuto Health Center IV |
| 59 | Kalisizo Hospital |
| 60 | Kambuga Hospital |
| 61 | Kamuli General Hospital |
| 62 | Kapchorwa Hospital |
| 63 | Kasangati Health Center IV |
| 64 | Katabi UPDF Hospital |
| 65 | Katakwi General Hospital |
| 66 | Kataraka Health Center IV |
| 67 | Kawolo Hospital |
| 68 | Kayunga Hospital |
| 69 | Kebisoni Health Center IV |
| 70 | Kibaale Health Center IV |
| 71 | Kiboga Hospital |
| 72 | Kida Hospital |
| 73 | Kidera Health Center IV |
| 74 | Kigandalo Health Center IV |
| 75 | Kigorobya Health Center IV |
| 76 | Kikyo Health Center IV |
| 77 | Kiryandongo Hospital |
| 78 | Kisubi Hospital |
| 79 | Kitagata Hospital |
| 80 | Kitgum Hospital |
| 81 | Kitojo Hospital |
| 82 | Kiwangala Health Center IV |
| 83 | Kiyumba Health Center IV |
| 84 | Koboko Hospital |
| 85 | Kotido Health Center IV |
| 86 | Kumi Health Center IV |
| 87 | Kyabugimbi Health Center IV |
| 88 | Kyankwanzi Health Center IV |
| 89 | Kyantongo Health Center IV |
| 90 | Kyazanga Health Center IV |
| 91 | Kyegegwa Health Center IV |
| 92 | Lalogi Health Center IV |
| 93 | Lubaga Hospital |
| 94 | Lwengo Health Center IV |
| 95 | Maddu Health Center IV |
| 96 | Madi Opei Health Center IV |
| 97 | Maracha Hospital |
| 98 | Masafu Hospital |
| 99 | Masindi Hospital |
| 100 | Midigo Health Center IV |
| 101 | Mitooma Health Center IV |
| 102 | Moyo Hospital |
| 103 | Mpigi Health Center IV |
| 104 | Mpumudde Health Center IV |
| 105 | Mukono Health Center IV |
| 106 | Mulanda Health Center IV |
| 107 | Mungula Health Center IV |
| 108 | Muyembe Health Center IV |
| 109 | Nabilatuk Health Center IV |
| 110 | Nakivale Health Center III |
| 111 | Namayumba Health Center IV |
| 112 | Namutumba Health Center III |
| 113 | Ndejje Health Center IV |
| 114 | Ngoma Health Center IV |
| 115 | Ngora Hospice |
| 116 | Ngora Hospital |
| 117 | Nsiika Health Center IV |
| 118 | Nsinze Health Center IV |
| 119 | Ntara Health Center IV |
| 120 | Ntusi Health Center IV |
| 121 | Ntwetwe Health Center IV |
| 122 | Nyakibale Hospital |
| 123 | Nyapea Hospital |
| 124 | Obongi Health Center IV |
| 125 | Padibe Health Center IV |
| 126 | Pajule Health Center IV |
| 127 | Pakwach Health Center IV |
| 128 | Pallisa Hospital |
| 129 | Peace Hospice |
| 130 | Princess Diana Health Center IV |
| 131 | Reach Out Mbuya |
| 132 | Rhino Camp Health Center IV |
| 133 | Rubongi Millitary Hospital |
| 134 | Rugazi Health Center IV |
| 135 | Ruhoko Health Center IV |
| 136 | Rushere Hospital |
| 137 | Rwashamaire Health Center IV |
| 138 | Rwesande Health Center IV |
| 139 | Serere Health Center IV |
| 140 | St. Anthony Hospital |
| 141 | St. Claire Orungo Health Center III |
| 142 | St. Francis Hospital Mutolere |
| 143 | St. Francis Hospital Naggalama |
| 144 | St. Francis Hospital Nkokonjeru |
| 145 | St. Francis Hospital, Buluba |
| 146 | St. Joseph's Kitgum Hospital |
| 147 | UPDF Rehabilitation Hospital |
| 148 | Wakiso Health Center IV |
| 149 | Yumbe Hospital |
