# Supplementary material for: Palliative care in Uganda: quantitative descriptive study of key palliative care indicators 2018-2020
Source: BMC Palliat Care. 2022 Apr 22;21:55. doi: 10.1186/s12904-022-00930-7 (PMC9023726; doi:10.1186/s12904-022-00930-7)
Supplement: Supplementary file 2 — Additional file 2. Average monthly volumes (ml) of green morphine distributed to mHealth surveillance survey facilities in 2019. List of detailed morphine volumes by month. [file 12904_2022_930_MOESM2_ESM.docx]

Additional File 2

Average monthly volumes (ml) of green morphine distributed to mHealth surveillance survey facilities in 2019

| **Month** | **Public** | **Private** |
| --- | --- | --- |
| Jan | 4800 | 6975 |
| Feb | 34800 | 23122 |
| Mar | 1200 | 0 |
| Apr | 8400 | 8195.12 |
| May | 900 | 83243.9 |
| Jun | 1500 | 16670.7 |
| Jul | 56700 | 15426.8 |
| Aug | 17100 | 33097.6 |
| Sep | 51900 | 2122 |
| Oct | 17700 | 11865.9 |
| Nov | 29400 | 22097.6 |
| Dec | 3600 | 28878.1 |
| **2019** | **228000** | **251695** |
