# Supplementary material for: Palliative care in Uganda: quantitative descriptive study of key palliative care indicators 2018-2020
Source: BMC Palliat Care. 2022 Apr 22;21:55. doi: 10.1186/s12904-022-00930-7 (PMC9023726; doi:10.1186/s12904-022-00930-7)
Supplement: Supplementary file 3 — Additional file 3. Districts with no accredited palliative care facility as of January 2020. List of districts with no accredited palliative care facilities as of January 2020. [file 12904_2022_930_MOESM3_ESM.docx]

Additional File 3

Districts with no accredited palliative care facility as of January 2020

|  | **District** | **Region** |
| --- | --- | --- |
| 1 | Buvuma | Central |
| 2 | Kalangala | Central |
| 3 | Kassanda | Central |
| 4 | Lyantonde | Central |
| 5 | Nakasongola | Central |
| 6 | Ssembabule | Central |
| 7 | Budaka | Eastern |
| 8 | Bugweri | Eastern |
| 9 | Bulambuli | Eastern |
| 10 | Kaberamaido | Eastern |
| 11 | Kalaki | Eastern |
| 12 | Kapelebyong | Eastern |
| 13 | Kween | Eastern |
| 14 | Luuka | Eastern |
| 15 | Namayingo | Eastern |
| 16 | Namisindwa | Eastern |
| 17 | Alebtong | Northern |
| 18 | Amudat | Northern |
| 19 | Dokolo | Northern |
| 20 | Karenga | Northern |
| 21 | Nakapiripirit | Northern |
| 22 | Otuke | Northern |
| 23 | Pader | Northern |
| 24 | Bunyangabu | Western |
| 25 | Kamwenge | Western |
| 26 | Kazo | Western |
| 27 | Kyenjojo | Western |
| 28 | Ntoroko | Western |
| 29 | Rubanda | Western |
| 30 | Rukiga | Western |
