# Supplementary material for: Palliative care in Uganda: quantitative descriptive study of key palliative care indicators 2018-2020
Source: BMC Palliat Care. 2022 Apr 22;21:55. doi: 10.1186/s12904-022-00930-7 (PMC9023726; doi:10.1186/s12904-022-00930-7)
Supplement: Supplementary file 4 — Additional file 4. Number of people living in districts with palliative care facilities by Uganda’s subregions (2014 census). List of Uganda’s subregions and number of people living in districts by number of accredited palliative care facilities, indicating the need in palliative care services in number of people. [file 12904_2022_930_MOESM4_ESM.docx]

Additional File 4

Number of people living in districts with palliative care facilities by Uganda’s subregions (2014 census)

| **Subregion** | **0 facilities** | **1 facility** | **2 to 4 facilities** | **5 or more facilities** | **Total population** |
| --- | --- | --- | --- | --- | --- |
| Acholi | 0 | 886730 | 614032 | 0 | 1500762 |
| Central | 620163 | 1870602 | 3147081 | 3801502 | 9439348 |
| East Central | 708239 | 1186332 | 1875088 | 988322 | 4757981 |
| Elgon | 472569 | 393871 | 880925 | 0 | 1747365 |
| Karamoja | 245283 | 719727 | 0 | 0 | 965010 |
| Lango | 514888 | 328479 | 1178180 | 0 | 2021547 |
| Southwestern | 474676 | 919404 | 1734886 | 1143439 | 4272405 |
| Teso | 302606 | 1635602 | 678020 | 0 | 2616228 |
| West Nile | 0 | 1069947 | 1590719 | 0 | 2660666 |
| Western | 659456 | 2024353 | 1659804 | 298989 | 4642602 |
| **All Uganda** | **3997880** | **11035047** | **13358735** | **6232252** | **34623914** |
