# Supplementary material for: Palliative care in Uganda: quantitative descriptive study of key palliative care indicators 2018-2020
Source: BMC Palliat Care. 2022 Apr 22;21:55. doi: 10.1186/s12904-022-00930-7 (PMC9023726; doi:10.1186/s12904-022-00930-7)
Supplement: Supplementary file 5 — Additional file 5. Other cancers reported by the mHealth surveillance survey participating facilities. Types of cancer of patients seeking palliative care services. [file 12904_2022_930_MOESM5_ESM.docx]

Additional File 5

Other cancers reported by the mHealth surveillance survey participating facilities

|  | **Condition** | **% of other cancers** |
| --- | --- | --- |
| 1 | Sarcomas | 10.6% |
| 2 | Lymphoma (including Hodgkin's, non-Hodgkin's, Burkitt's) | 6.8% |
| 3 | Gynecological cancers (ovarian, cervical, vulvar, endometrium, uterine) | 6.3% |
| 4 | Stomach cancer | 4.6% |
| 5 | Rectal cancer | 4.3% |
| 6 | Lung cancer | 3.8% |
| 7 | Penile cancer | 3.8% |
| 8 | Kidney cancer | 3.7% |
| 9 | Leukemia | 3.5% |
| 10 | Pancreatic cancer | 3.4% |
| 11 | Osteosarcoma | 3.2% |
| 12 | Multiple myeloma | 2.9% |
| 13 | Abdominal tumor | 2.6% |
| 14 | Squamous cell carcinoma | 2.6% |
| 15 | Bladder cancer | 2.2% |
| 16 | Melanoma | 2.0% |
| 17 | Bone cancer | 1.5% |
| 18 | Nasopharyngeal carcinoma | 1.5% |
| 19 | Retinoblastoma | 1.4% |
| 20 | Brain tumor | 1.4% |
| 21 | Liver cancer | 1.2% |
| 22 | Oral cancer | 1.1% |
| 23 | Breast cancer | 0.9% |
| 24 | Skin cancer | 0.9% |
| 25 | Thyroid gland cancer | 0.8% |
| 26 | Adenocarcinoma | 0.8% |
| 27 | Colon cancer | 0.8% |
| 28 | Gastric cancer | 0.6% |
| 29 | Larynx cancer | 0.5% |
| 30 | Parotid gland cancer | 0.5% |
| 31 | Spinal bone cancer | 0.5% |
| 32 | Basal cell carcinoma | 0.5% |
| 33 | Cervical bone cancer | 0.3% |
| 34 | Chest tumor | 0.3% |
| 35 | Cholangiocarcinoma | 0.3% |
| 36 | Eye cancer | 0.3% |
| 37 | Germ cell tumor | 0.3% |
| 38 | Lipoma | 0.3% |
| 39 | Pelvic cancer | 0.3% |
| 40 | Prostate cancer | 0.3% |
| 41 | Testicular cancer | 0.2% |
| 42 | Throat cancer | 0.2% |
| 43 | Urethral cancer | 0.2% |
