# Supplementary material for: Palliative care in Uganda: quantitative descriptive study of key palliative care indicators 2018-2020
Source: BMC Palliat Care. 2022 Apr 22;21:55. doi: 10.1186/s12904-022-00930-7 (PMC9023726; doi:10.1186/s12904-022-00930-7)
Supplement: Supplementary file 6 — Additional file 6. Other conditions mentioned by mHealth surveillance survey participating facilities. List of other conditions like hypertension, arthritis, and pain, reported the most when seeking palliative care. [file 12904_2022_930_MOESM6_ESM.docx]

Additional File 6

Other conditions mentioned by mHealth surveillance survey participating facilities

|  | **Condition** | **% mentions** |
| --- | --- | --- |
| 1 | Hypertension | 9.0% |
| 2 | Arthritis | 8.1% |
| 3 | Pains | 5.4% |
| 4 | Congestive Cardiac Failure (CCF) | 5.4% |
| 5 | Liver cirrhosis | 3.6% |
| 6 | Heart disease | 3.6% |
| 7 | Tuberculosis | 2.7% |
| 8 | Neurological disease | 2.7% |
| 9 | Osteoporosis | 2.7% |
| 10 | Stroke | 2.7% |
| 11 | Ulcers | 1.8% |
| 12 | Wounds | 1.8% |
| 13 | Cerebral Cavernous Malformation (CCM) | 1.8% |
| 14 | Fractures | 1.8% |
| 15 | Gangrene | 1.8% |
| 16 | Meningitis | 1.8% |
| 17 | Osteomyelitis | 1.8% |
| 18 | Peptic Ulcer Disease (PUD) | 1.8% |
| 19 | Psychosocial issues | 1.8% |
| 20 | Pulmonary disease | 1.8% |
| 21 | Skin conditions | 1.8% |
| 22 | Spondylosis | 1.8% |
| 23 | Adenitis | 1.8% |
| 24 | Ameloblastoma | 0.9% |
| 25 | Anaemia | 0.9% |
| 26 | Asthma | 0.9% |
| 27 | Cerebrovascular Accident | 0.9% |
| 28 | Chest conditions | 0.9% |
| 29 | Chronic skin ulcers | 0.9% |
| 30 | Chronic skin ulcers | 0.9% |
| 31 | Cough | 0.9% |
| 32 | Dermatomyositis | 0.9% |
| 33 | Diarrhea | 0.9% |
| 34 | Disc prolapse | 0.9% |
| 35 | Fistula | 0.9% |
| 36 | Flu | 0.9% |
| 37 | Keloids | 0.9% |
| 38 | Kidney disease | 0.9% |
| 39 | Loss of appetite | 0.9% |
| 40 | Lumbago | 0.9% |
| 41 | Necrotizing fasciitis | 0.9% |
| 42 | Orchiectomy | 0.9% |
| 43 | Parkinson's syndrome | 0.9% |
| 44 | Pneumonia | 0.9% |
| 45 | Seizures | 0.9% |
| 46 | Septicemia | 0.9% |
| 47 | Spiritual issues | 0.9% |
| 48 | Stephenson syndrome | 0.9% |
| 49 | Stigma | 0.9% |
| 50 | Vomiting | 0.9% |
